# Assessing Insecticide Hazard to Bumble Bees Foraging on Flowering Weeds in Treated Lawns

**DOI:** 10.1371/journal.pone.0066375

**Published:** 2013-06-12

**Authors:** Jonathan L. Larson, Carl T. Redmond, Daniel A. Potter

**Affiliations:** Department of Entomology, University of Kentucky, Lexington, Kentucky, United States of America; Royal Holloway University of London, United Kingdom

## Abstract

Maintaining bee-friendly habitats in cities and suburbs can help conserve the vital pollination services of declining bee populations. Despite label precautions not to apply them to blooming plants, neonicotinoids and other residual systemic insecticides may be applied for preventive control of lawn insect pests when spring-flowering weeds are present. Dietary exposure to neonicotinoids adversely affects bees, but the extent of hazard from field usage is controversial. We exposed colonies of the bumble bee *Bombus impatiens* to turf with blooming white clover that had been treated with clothianidin, a neonicotinoid, or with chlorantraniliprole, the first anthranilic diamide labeled for use on lawns. The sprays were applied at label rate and lightly irrigated. After residues had dried, colonies were confined to forage for six days, and then moved to a non-treated rural site to openly forage and develop. Colonies exposed to clothianidin-treated weedy turf had delayed weight gain and produced no new queens whereas those exposed to chlorantraniliprole-treated plots developed normally compared with controls. Neither bumble bees nor honey bees avoided foraging on treated white clover in open plots. Nectar from clover blooms directly contaminated by spray residues contained 171±44 ppb clothianidin. Notably, neither insecticide adversely impacted bee colonies confined on the treated turf after it had been mown to remove clover blooms present at the time of treatment, and new blooms had formed. Our results validate EPA label precautionary statements not to apply neonicotinoids to blooming nectar-producing plants if bees may visit the treatment area. Whatever systemic hazard through lawn weeds they may pose appears transitory, however, and direct hazard can be mitigated by adhering to label precautions, or if blooms inadvertently are contaminated, by mowing to remove them. Chlorantraniliprole usage on lawns appears non-hazardous to bumble bees.

## Introduction

Native bee and honey bee populations are declining due to habitat loss and fragmentation, disease, and other stresses [1]–[5]. Bees in cities and suburbs survive by gathering nectar and pollen from flowering plants in lawns, gardens, and patches of semi-natural habitat [2]–[6]. In the United States, where about one million hectares of farmland and natural habitat are converted to urban areas each year [6], turf grasses now cover about 164,000 km^2^, an area three times larger than any agricultural crop [7]. Most (>75%) of that turf is comprised of residential, commercial, and institutional lawns, many of which are treated with insecticides by homeowners or commercial lawn care providers [8], [9].

Neonicotinoids, systemic insecticides that move via sap throughout treated plants, are potent selective agonists of nicotinic acetylcholine receptors in insects [10]. Imidacloprid, clothianidin, and thiamethoxam are widely used on lawns [8]. Typically applied as sprays or granules in spring and leached into the soil by irrigation or rainfall, they provide several months of residual control of root-feeding grubs and other pests [8]. Despite label precautions stating not to apply neonicotinoids to plants in bloom, applications are sometimes made when lawn weeds such as dandelions and white clover are flowering. These weeds are attractive to native pollinators, especially bumble bees, and to managed and feral honey bees [11]–[13].

Although residue levels in nectar and pollen of neonicotinoid-treated crops tend to be below acute toxicity levels for bees [14]–[16], lethal and sublethal effects of dietary exposure including impaired learning, memory, and navigational abilities of honey bees [5], [16]–[19] and reduced foraging, colony growth, and queen production by bumble bees [20]–[23] have been described. Most of the evidence, however, comes from studies in which doses of the insecticide were lab-fed to bees in sugar water or pollen, and in some such trials, dosages typical of those found in seed-treated crops had no apparent adverse effects 24,25. Some field studies in which bees were exposed to crops grown from neonicotinoid-treated seeds failed to detect detrimental effects on colony health [26], [27]. Bumble bee colonies exposed to dry spray residues of imidacloprid on weedy turf gained less weight and produced fewer workers, brood chambers, and honey pots compared to controls, but when spray residues were watered into the soil, or the insecticide was applied in granular form, no adverse effects on those measures of colony health were observed [12]. The extent to which trace dietary neonicotinoids impact bees in field settings remains controversial and requires studies with relevant exposure and duration to resolve [16], [28].

Anthranilic diamides are a relatively new class of insecticides that activate insect ryanodine receptors by stimulating release of calcium stores from muscle cells causing lethal paralysis in sensitive species [29]. They have low vertebrate toxicity, low use rates, and 3–5 month residual activity in soil, as well as low impact on non-target invertebrates [29]–[31]. Chlorantraniliprole, the first anthranilic diamide lawn insecticide, received reduced-risk status from the US Environmental Protection Agency [8]. Compared to neonicotinoids, it has similar efficacy against root-feeding scarab grubs and weevil larvae, better activity against caterpillar pests, but is less active against chinch bugs [8]. Chlorantraniliprole has low acute bee toxicity [29] but its potential reproductive effects on bees with realistic field exposure have not been evaluated. If benign, it and other anthranilic diamides could be a more bee-friendly option for insect control in lawns, gardens, and other settings where bees are active.

We exposed colonies of the bumble bee *Bombus impatiens* to turf intermixed with white clover where clothianidin or chlorantraniliprole had been applied at label rates to test the hypothesis that the latter is relatively less hazardous to colonies foraging on flowering weeds in treated lawns. Several scenarios were used to assess the insecticides' respective impacts on colony health and queen production. Our results showed that colonies foraging on the neonicotinoid-treated turf had higher worker and brood mortality, reduced honey pot production, delayed weight gain, and impaired queen production compared to controls, but also suggested that the hazard is reduced after blooms present at the time of application are removed by mowing. The anthranilic diamide appears to be non-hazardous to bumble bees even when used on lawns where flowering weeds are present.

## Results

**Figure 1:**
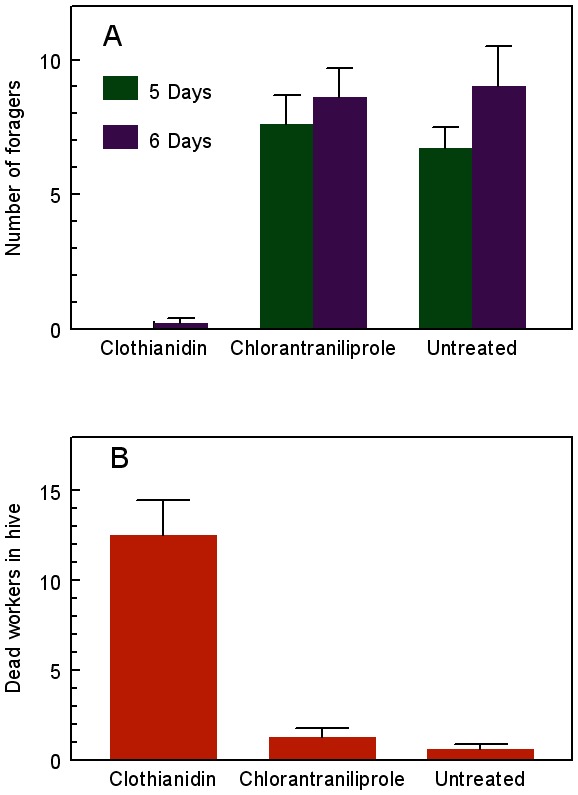
Foraging and dead workers during exposure to treated turf. Mean (±SE) numbers of (A) bees foraging in enclosures during two mid-afternoon inspections on the 5th and 6th days, and (B) dead non-callow workers observed in hives on the 6th day of exposure of bumble bee colonies to weedy lawn turf with residues of a neonicotinoid (clothianidin) or anthranilic diamide (chlorantraniliprole) applied at label rates. For foragers, clothianindin<chlorantraniliprole = untreated on both census dates; for dead workers, clothianidin>chlorantraniliprole = untreated (Friedman tests, P<0.001).

**Figure 2:**
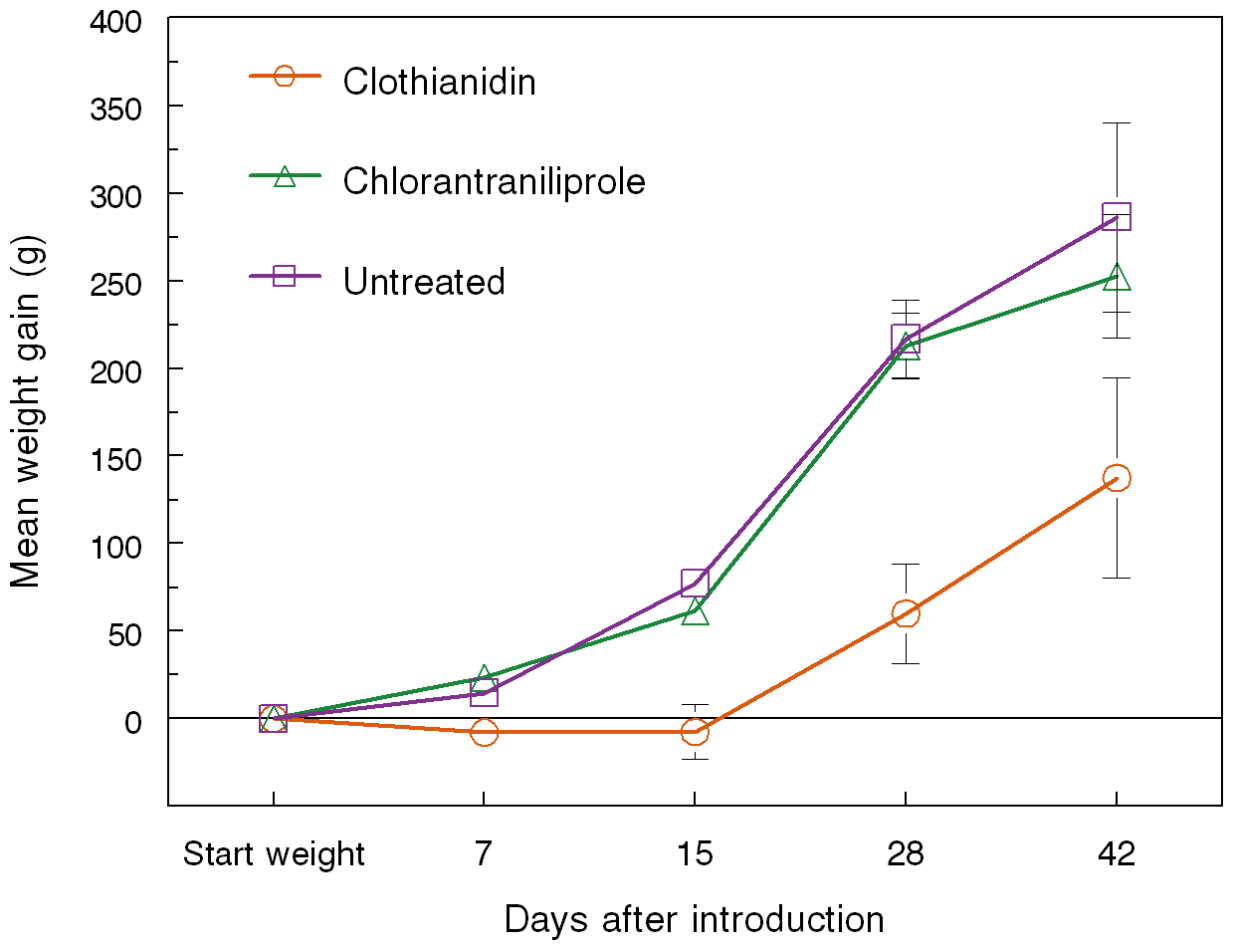
Colony weight change following exposure to treated turf. Mean (± SE) weight change (g) of *Bombus impatiens* colonies (10 per treatment) after foraging 6 days on insecticide-treated lawn turf with white clover and then being moved to an insecticide free site to openly forage for another 6 weeks (Repeated measures ANOVA: *F*_2,90_ = 14.8, *P*<0.001; *F*_4,90_ = 45.1, *P*<0.001; *F*_8,90_ = 2.2, *P*<0.05 for treatment, date, and treatment×date interactions, respectively). Clothianidin-exposed colonies lagged behind the others on all dates (*F*_2,18_ = 6.5, 15.6. 12.7, 3.1; *P*<0.01, 0.001, 0.001, 0.07 at 7, 15, 28, and 42 days after introduction, respectively.

**Figure 3:**
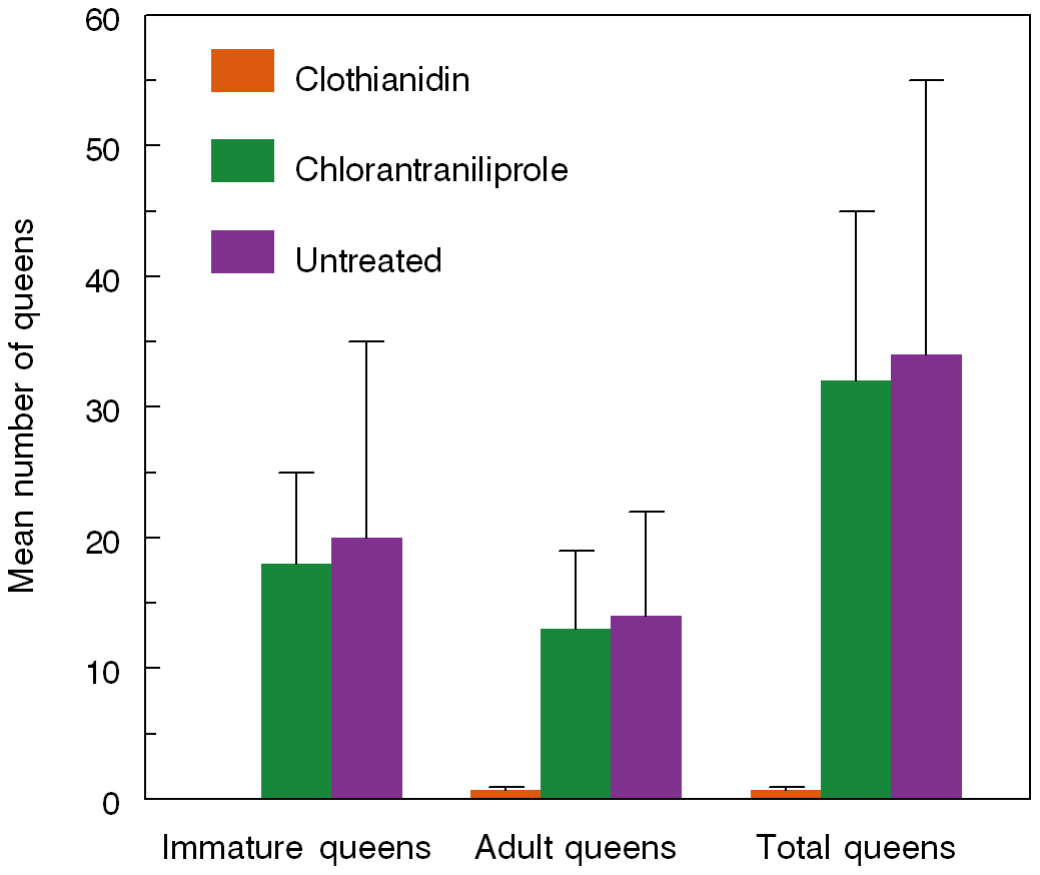
Queen production following exposure to treated turf. Mean (+ SE) numbers of queens produced by *Bombus impatiens* colonies that foraged for 6 days on insecticide-treated lawn turf with white clover and then were moved to an insecticide-free site to openly forage another 6 weeks (Friedman tests: Immature queens, P = 0.03; Adult queens, P = 0.08; Total queens, P = 0.05. Numbers of colonies (out of 10) that produced new queens were 0, 7, and 6 for clothianidin, chlorantraniliprole, and untreated hives, respectively. For the subset of colonies that produced new queens, those exposed to chlorantraniliprole-treated or untreated weedy turf produced similar numbers of immature, adult, and total queens (Kruskal-Wallis test, P = 0.69, 0.84, 0.95, respectively). Queens present in clothianidin exposed colonies likely represent the original mother queen.

Colonies exposed to clothianidin-treated weedy turf showed reduced foraging activity and increased worker mortality in the hives within five days (Fig. 1). They also gained weight more slowly after being moved to an insecticide-free site where they were left to openly forage for six more weeks (Fig. 2). Although statistically significant differences were no longer detected by analysis of variance by the time the hives were dissected, there remained consistent trends for fewer live adults (workers and males), honey pots, and reduced colony weight of clothianidin-exposed colonies compared to the controls (*P* = 0.052, 0.09, 0.058, respectively; pre-planned linear contrasts, Table 1). More importantly, clothianidin- exposed colonies failed to produce new queens (Fig. 3). Chlorantraniliprole-exposed colonies showed no impairment in weight gain or reductions in other indicators of colony health, including new queen production, compared to the controls (Fig. 3, Table 1),

**Table 1:** Condition of *Bombus impatiens* colonies that had been exposed to insecticide-treated turf with flowering white clover for 6 days, after which they were moved to an insecticide-free site to openly forage for 6 weeks before this evaluation.^a^

	Adults (workers and males) per hive			Immatures per hive^b^				
Treatment	Live	Dead	% dead	Live	Dead	Honey pots	Total wt (g) of live adults^c^	Hive wt (g)
Clothianidin	173±39	33±7	31.8±11.1	84±15	9±3	36±12	28.2±6.9	709±59
Chlorantraniliprole	199±31	35±14	17.4±7.3	45±10	18±9	51±10	31.4±4.8	826±35
Untreated	271±30	54±16	18.2±5.9	65±14	27±13	77±22	42.9±5.6	857±56

The turf was lightly irrigated after insecticide application; the surface had thoroughly dried before bees were introduced.

a Data are means (± SE). ANOVA (df = 2, 18): live, *F* = 2.31, *P* = 0.13; dead, *F* = 0.92, *P* = 0.42; % dead, *F* = 0.93, *P* = 0.41; wt live adults, *F* = 1.8, *P* = 0.19; live immature, *F* = 2.45, *P* = 0.12; dead immature, *F* = 0.90, *P* = 0.42, honey pots, *F* = 2.31, *P* = 0.13, hive wt, *F* = 2.27, *P* = 0.13. *P*-values from pre-planned linear contrasts between clothianidin versus untreated were 0.053, 0.23, 0.27, 0.28, 0.20, 0.09, 0.09, and 0.058, respectively. For chlorantraniliprole versus untreated, they were 0.15, 0.29, 0.95, 0.29, 0.51, 0.27, 0.18, and 0.67, respectively.

b larvae, pupae, and fully-formed workers and males still enclosed in the pupal exoskeleton within the cell.

c adult workers, males, and queens.

Nectar extracted by centrifugation from 100-flower samples of clover flowers from the clothianidin-treated plots one week after application in 2012 contained 171±44 ppb clothianidin (mean ± SE; range 89–319; n = 5), whereas nectar samples from flowers in open, non-treated areas contained no detectable insecticides. Nearly all of the flowers under the enclosures on non-treated or chlorantraniliprole-treated plots had been pollinated which precluded collecting sufficient nectar from them for analysis.

In another set of trials, *B. impatiens* colonies evaluated after two weeks' exposure to clothianidin-treated turf with flowering white clover suffered significantly higher worker and brood mortality and produced fewer honey pots, whereas colonies similarly exposed to plots that had been treated with chlorantraniliprole showed no adverse effects compared to the untreated controls (Table 2). Notably, neither insecticide adversely affected a second set of colonies introduced into the enclosures after the turf had been mown to remove the original flower heads, and new flowers had formed (Table 3). Hives that had been confined on chlorantraniliprole-treated turf in fact had significantly higher numbers of live adult workers than did the untreated controls (two-tailed Dunnett's test, *P* = 0.02; Table 3).

**Table 2:** Condition of *Bombus impatiens* colonies that were evaluated immediately after being exposed to insecticide-treated turf with flowering white clover for 2 wk.

	Adult workers per hive^a^		Immatures per hive^b^				
Treatment	Live	Dead	Live	Dead	Honey pots	Total weight (g) of live adults^c^	Hive weight (g)
Clothianidin	59±12*	26±5*	21±8	13±2*	33±5*	7.7±1.4*	580±17
Chlorantraniliprole	99±12	6±2	31±9	4±1	47±5	12.2±1.5	599±11
Untreated	106±8	7±3	17±10	4±1	51±4	12.8±1.6	602±6

Plots were treated June 1; bee colonies were introduced the following day.

Data are means (± SE). ANOVA (df = 2, 22): live, *F* = 4.57, *P*<0.05; dead, *F* = 9.88, *P*<0.01; wt live workers, *F* = 3.46, *P* = 0.05; live immature, *F* = 0.57, *P* = 0.57; dead immature, *F* = 9.25, *P*<0.01; honey pots, *F* = 3.56, *P*<0.05; hive wt, *F* = 0.69, *P* = 0.51;

* denotes means significantly higher or lower than colonies on untreated turf (Dunnett's test, α = 0.05).

a All adults (other than original queen) were workers as there would not have been time for males to emerge from the brood (K. Skyrm, Koppert Biological Systems, *personal communication*).

b larvae, pupae, and fully-formed workers still enclosed in the pupal exoskeleton within the cell.

c adult workers and original queen.

**Table 3:** Absence of acute adverse effects on *Bombus impatiens* colonies after 2 weeks' exposure to turf with flowering white clover that had bloomed after the sward was mown to remove flowers present at the time of treatment.

	Adult workers per hive^a^		Immature bees per hive^b^				
Treatment	Live	Dead	Live	Dead	Honey pots	Total weight (g) of live adults^c^	Hive weight (g)
Clothianidin	93±9	11±4	12±8	6±1	52±6	13.0±1.3	585±11
Chlorantraniliprole	130±12*	7±2	8±4	6±2	69±6	16.7±1.6	621±16
Untreated	81±8	7±2	0	3±1	56±3	11.3±0.9	588±8

Insecticide application, mowing, and introduction of bee colonies were on June 1, 15, and 22, respectively.

ANOVA (df = 2, 19): live, *F* = 6.01, *P* = 0.02; dead, *F* = 1.05, *P* = 0.37; Wt live workers, *F* = 3.31, *P* = 0.08; live immature, *F* = 1.60, *P* = 0.25; dead immature, *F* = 0.54, *P* = 0.6, honey pots, *F* = 2.15, *P* = 0.17, hive wt, *F* = 1.93, *P* = 0.20.

* Significantly higher than untreated; 2-tailed Dunnett's test.

a All adults (other than original queen) were workers as there would not have been time for males to emerge from the brood (K. Skyrm, Koppert Biological Systems, *personal communication*).

b larvae, pupae, and fully-formed workers still enclosed in the pupal exoskeleton within the cell.

c adult workers and original queen.

**Figure 4:**
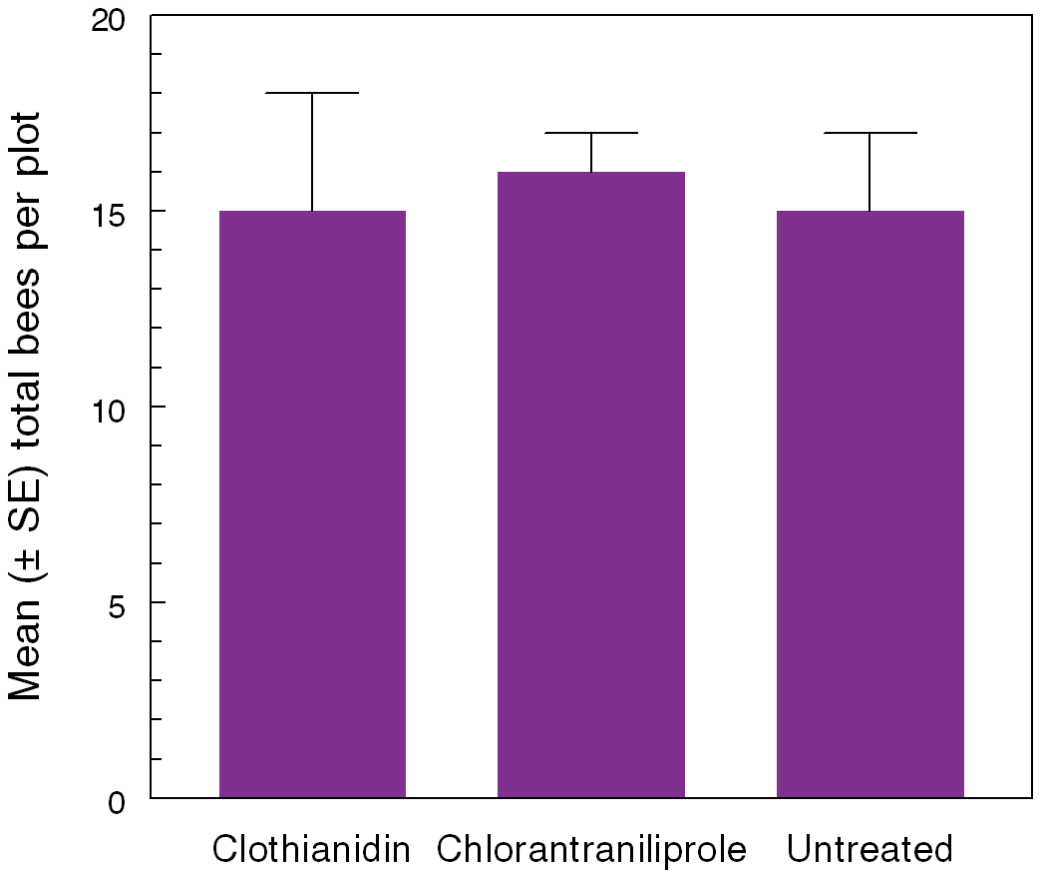
Non-avoidance of treated turf by bees. Bumble bees and honey bees did not discriminate between weedy lawn turf with or without non-irrigated insecticide residues (*F*_2, 8_ = 0.02, *P* = 0.98). Plots were treated at label rate with residues left on the surface (not watered in) and a walk-through count of foragers on the intermixed white clover was taken on seven successive days. Data shown are mean (+ SE) totals of both types of bees. Means for bumble bees were 6.4±1.1; 6.6±0.9, 5.8±1.2 (*F*_2, 8_ = 0.16, *P* = 0.85); means for honey bees were 8.8±1.5, 9.0±0.7, 9.4±0.5 (*F*_2, 8_ = 0.10, *P* = 0.90) for clothianidin-, chlorantraniliprole-, and non-treated plots, respectively.

Neither bumble bees nor honey bees avoided foraging on white clover in turf that had been treated with either insecticide. Similar numbers of bumble bees, honey bees, and total bees were observed on clover blooms on each set of plots (Fig. 4).

## Discussion

This study shows with field exposure that clothianidin, a representative neonicotinoid, has the potential to impair queen production by bumble bee colonies foraging for less than a week on flowering weeds in recently-treated lawns. United States Environmental Protection Agency (EPA) label precautionary statements specify not to apply clothianidin, or other neonicotinoids, to blooming nectar-producing plants if bees are visiting the treatment area, but such exposures nevertheless may occur, especially when lawns are treated in spring for preventive grub control. Our results validate those EPA label precautions. They also confirm the results of other recent studies that showed acute mortality and impaired queen production when bees ingested neonicotinoid-spiked food [21]–[23], demonstrating similar effects from a plausible field exposure. Notably, no adverse effects were seen on bee colonies exposed to residues of chlorantraniliprole, a selective ryanodine receptor agonist, under the same conditions.

The concentrations of clothianidin we detected in clover nectar are higher than those that typically occur from systemic transfer of neonicotinoids into nectar of seed-treated crops [5], [14], [15], and also much higher than lab-fed oral dosages of imidacloprid shown to adversely affect individual and colony-level traits, including reproduction, in bees [19]–[23], [28]. A literature search found nothing on spatial or temporal translocation of neonicotinoids from roots into nectar or pollen of clover or similar small plants. Thus, while we can suggest several plausible ways that a lawn spray application might contaminate such nectar, the precise mechanisms by which it occurred in our study remain largely unknown.

The equipment with which we applied the insecticides, a lawn care spray gun and a multiple-nozzle boom sprayer, delivered similar pressure, droplet size, and spray volume as sprayers used in the turf care industry. It is likely that the sprays directly contaminated the nectar, which in non-pollinated *T. repens* florets is retained at the floret base for at least a week with no decrease in quantity or sugar content until pollination or senescence [32]. Clothianidin may also have been systemically translocated through foliage. Also, the numerous densely-arranged individual florets of not-yet-opened flower heads may have sufficient surface area shielded from UV light to allow translocation through cells of the nectary walls before such residues deteriorate. Although the turf was irrigated immediately after the insecticides were applied, some residues may have remained on the clover petals and leaves, and on the turfgrass, so that foraging bees were exposed both through contact and ingestion.

Neonicotinoids are mainly acropetally transported in the xylem [15], [16], [33]. Given clothianidin's prolonged (>9 month) half-life from field dissipation in soil [34], it is unlikely that, in just three weeks, degradation of residues in the root zone can explain the lack of acute effects on bees foraging on clover that bloomed after mowing. Clothianidin is the least water-soluble neonicotinoid used on turf [34]. Sorption of neonicotinoids to soil organic components reduces the amount that is translocated [33], [35]. Translocation is driven by transpiration and plant growth, processes likely to be greater for foliage than for floral tissues and nectar. Neonicotinoid uptake via roots typically deposits the highest concentrations in the oldest foliage, with limited mobilization from mature to new leaves 33,35, so in a mixed stand of turfgrass and flowering weeds, the competing grass could possibly act as a sink until being removed by mowing.

Clearly, more needs to be known about the movement and longevity of surface-applied neonicotinoids in clover and other small flowering plants to better interpret our results. Nevertheless, the results of our trial in which colonies were confined on treated weedy turf before or after the stand had been mowed, and earlier work showing absence of acute effects on bumble bees when a granular formulation of imidacloprid was applied to weedy turf and watered in [12], suggest that once the residues are leached into the soil by watering or rainfall, translocation via the roots is unlikely to pose a prolonged systemic hazard to bees.

Neither bumble bees nor honey bees avoided foraging on flowering clover contaminated with residues of clothianidin or chlorantraniliprole. That finding is consistent with previous studies showing bumble bees' non-avoidance of flowering clover in lawn grass that had been sprayed with imidacloprid [12], and bees' ready ingestion of syrup or plant guttation water containing toxic levels of neonicotinoids [22], [36]. Thus, worker bees from colonies in non-treated landscapes may be exposed to insecticide residues when foraging on treated lawns. If such bees acquire a lethal dose they will not return to the colony, reducing its workforce. Even sublethal neonicotinoid exposure can impair workers' foraging efficiency, leading to food shortage and decreased colony success [22]. Workers that bring contaminated nectar or pollen back to the colony could potentially affect development and survival of nest-mates. Bumble bee colonies are annual and only the new queens produced will survive the winter. In the spring, when queens are foraging and subsequently when colonies are small and contain only a few workers, they may be especially vulnerable to insecticide exposure [2], [22]. Typically only the largest colonies succeed in producing queens [37]–[39].

It is possible, had we not sacrificed them, that clothianidin-exposed colonies could have recovered from the initial stress and produced queens later in the summer or autumn. However, any delay in switching from worker to queen production increases the chances of colony failure due to pathogens, predators, weather-related stress, or other factors. Moreover, queens produced later in the growing season are less likely to survive than are earlier-produced queens [37]–[39]. Without timely investment in reproductive output, the potential loss of queen production due to neonicotinoid exposure could lead to lower local populations of bumble bees over successive years.

Besides mowing to remove flower heads before or immediately after application, bee exposure to pesticide residues on lawns could be reduced by controlling flowering weeds with herbicides or by delaying applications until after bloom of spring-flowering weeds. Such practices, however, may be difficult to ensure or may not always be practical, especially in high-volume commercial lawn care [8].

Anthranilic diamides, including chlorantraniliprole, show high selectivity for insect ryanodine receptors (RyRs) when compared to mammalian RyRs [29], [40]. Chlorantraniliprole is active against caterpillars and some dipteran and coleopteran pests, mainly by ingestion and secondarily by contact [29], [30]. It appears to have little or no activity against predatory, parasitic, and social wasps, solitary and social bees, and ants [29]–[31]. The basis for that selectivity is not yet understood but may involve differences in channel properties between RyRs of sensitive species and those of the aforementioned types of Hymenoptera [40].

Bumble bees and other native bees provide pollination services to urban and suburban gardens and landscapes [2]–[5], [13]. With their populations imperiled by habitat loss, diseases, parasites, and other stresses, reducing hazards posed to them by insecticides is important [1]–[5]. When neonicotinoids are applied to lawns, systemic hazard to bees through flowering weeds appears to be transitory and direct hazard can be mitigated by strict adherence to label precautions, or if blooms inadvertently are contaminated, by mowing to remove them. Chlorantraniliprole appears to be a good fit for industry initiatives to reduce the impacts of turf and landscape management on pollinators.

## Materials and Methods

### Insecticide impacts on foraging, colony health and queen production

This trial evaluated the scenario of resident bees foraging on flowering weeds in a newly-treated lawn for six days before the turf was mowed. The exposure phase was done at the A.J. Powell Turf Research Center, University of Kentucky, near Lexington, KY in a 1-ha sward of Kentucky bluegrass (*Poa pratensis* L.) with about 30% cover (by visual estimate) of flowering white clover (*Trifolium repens* L.). Plots (3.35×3.35 m; 10 replicates of each insecticide) were situated on areas with similar clover density and were at least 2 m apart. Treatments were clothianidin (Arena 50 WDG; Valent, Walnut Creek, CA), chlorantraniliprole (Acelepryn, 18.4% active ingredient (AI); Dupont, Wilmington, DE), and the untreated check. Both products were applied as they would be for scarab grub control at their high label rates, 0.45 and 0.23 kg AI ha^−1^ for clothianidin and chlorantraniliprole, respectively. The applications were made on 14 May 2012. We used a portable CO_2_ spray tank (R and D Sprayers, Opelousas, LA) equipped with a 1.8 m handheld boom with four Spraying System 8004 Tee Jet nozzles (Spraying Systems, Wheaton, IL) that delivered a pressure of 2109 g cm^−2^. Spray volume was 468 L ha^−1^, applied by making two passes in opposite directions over each plot. Separate spray bottles were used for each treatment. Residues were lightly watered in (30.3 liters per plot) from sprinkling cans about 1 h after application.

Screen enclosures (3.05×3.05 m; Instant Screen Shelters, Coleman; Wichita, KS) were erected on each plot 24 h after application. Commercial *Bombus impatiens* colonies (Research Mini-hives; Koppert, Howell, MI), one per enclosure (10 per treatment), were randomly assigned to the treatments after being blocked by their initial weight. Each colony was housed within a plastic hive within an outer cardboard box and started with 20 workers and a fertilized queen. Colonies were shipped with a syrup food sack which was left in the hives while they were confined on the weedy turf plots but removed when the bees were moved to the safe foraging site (see below). Colonies were introduced to the enclosures on 16 May, two days after the insecticides had been applied. Each enclosure was inspected in mid-afternoon on the 5th and 6th day after introduction and workers foraging within the enclosed area at that time were counted. After six days the doors of foraging the hive doors were closed at night, after workers had returned. Nest materials cannot be removed from the inner plastic hive without causing severe disturbance so they were weighed together. Hives were weighed after closure (23 May), replaced in their boxes, and then transported 12 km to Gainesway Farm (Lexington, KY) a 700 ha working horse farm at which no insecticides are applied to the pastures, grounds, or trees. The colonies were placed on concrete blocks at least 3 m apart along the edge of a woodlot. Their openings faced a pasture with wildflowers including clover. The site was at least 1 km from the nearest edge of the farm. Gainesway Farm is surrounded by pastures of other horse farms where no pesticides are applied, making it highly unlikely that foraging workers would be exposed to additional insecticides.

Colonies were left to openly forage at the horse farm site for 6 more weeks. They were inspected and weighed in the field on 31 May and 13 June. They were closed on 3 July, brought to the lab, and held at 4.4°C until evaluated. Colonies were weighed and then dissected, by replicate, over the following 1.5 weeks to assess numbers of living and dead adults (combined workers and males), queens, honey pots, and living and dead larvae and pupae, and weights of live adults and queens.

Samples of 100 presumably non-pollinated flowers (i.e., lacking drooped brown basal florets indicative of having been pollinated) were collected from each of five replicates of the clothianidin-treated plots after the bees were removed on the 6th day after treatment. Because nearly all blooms in chlorantraniliprole and control plots appeared to be pollinated, samples of 100 non-pollinated flowers were collected from each of five distinct untreated areas outside the enclosures but in the same turf sward. Florets were trimmed with scissors and then whole individual flowers were inverted and spun in individual 15 ml centrifuge tubes for 10 min at 2000 rpm to extract the nectar. Nectar samples (about 300 mg per 100 flowers) were consolidated within each plot, transferred to micro-centrifuge tubes, and sent to the USDA-AMS National Science Laboratory (Gastonia, NC) where they were analyzed for clothianidin residues (1 ppb level of detection) by liquid chromatography separation coupled with a tandem mass selective detection system (LC/MS/MS) following a modified version of the AOAC official method of analysis 2007.06 (QuEChERS method) [41].

### Acute effects of exposure to insecticide residues before and after mowing

This study was done on a different part of the same sward used for the exposure phase of the previously-described trial, using similar methods, except as follows. The treatments were made by a professional care applicator, supervised by the authors, on 1 June 2011. The insecticides were diluted in water and applied with a lawn spray gun (model 11-857-00 Mag 2000; 7.6 liters/min nozzle; GNC Industries, Pocahontas, AR) powered by an electric pump (FloJet model 4300-405; FloJet, Irvine, CA) at their label rates for grub control. Spray volume was 410 L ha^−2^. Residues were either allowed to dry on the surface, simulating what typically occurs with commercial lawn applications, or were watered in as described earlier. The open-bottom screened enclosures were erected on each plot 24 h later, and a commercial *B. impatiens* colony consisting of 20 workers and a fertilized queens as described above, was introduced to each enclosure that evening. Colonies were left to forage in the enclosures for two weeks before the hives were closed and brought to the lab for evaluation. The sward then was mowed (2.5 cm cutting height) to remove clover flowers present at the time of treatment. One week later (22 June), after new blooms had formed, another set of freshly-shipped bee colonies (Research Mini-hives; Koppert, Howell, MI) of the same size and age as those used for the initial challenge was introduced and left to forage in the enclosures for two weeks, after which the hives were closed and brought to the lab for evaluation. Initially there were five replicates for each combination of insecticide and watering regime, plus untreated controls, but because the irrigation main effect was non-significant for all dependent variables, data from irrigated and non-irrigated plots were combined for analysis.

### Avoidance

This study was done on a 2.5 ha sward of non-irrigated Kentucky bluegrass intermixed with white clover at the University of Kentucky's intramural sport field complex. Plots (3.7×3.7 m) were treated with clothianidin or chlorantraniliprole at label rates on 23 May 2012, using the portable CO_2_ sprayer described earlier. Those treatments, plus untreated plots, were arranged in a randomized complete block with five replicates per treatment (i.e. 15 plots in total). Untreated borders (2.44 m) surrounded each plot. Residues were not watered in, and there was no rainfall during the trial. Bee counts were taken daily for 1 week between 10:30 and 16:00 by slowly walking around each plot, staying in the border, and counting honey bees (*Apis mellifera*) and bumble bees (*Bombus* spp.) foraging on the clover. Each plot was observed for 1 minute, and after all plots had been inspected, the census was repeated, starting at the first plot, providing two counts within a 45-minute period. Bees moved from plot to plot, and between border areas and plots, so each count represented a snapshot of bees on a plot at that time.

### Statistical Analyses

Numbers of foraging workers in field enclosures, final colony weights, and parameters measured during dissections were compared among treatments by analysis of variance (ANOVA), followed by pre-planned linear contrasts to compare each of the individual insecticides to the untreated control. We used the angular transformation for percentages and square root or log transformations for those data sets where treatment variances were non-homogeneous. Non-parametric tests were used for number of new queens where ANOVA assumptions were not met. Colony weights over time were compared using repeated measure ANOVAs. Counts of bees observed in the avoidance trial plots were totaled across census dates and analyzed by two-way ANOVA. All data are given as original means ± SE. Statistix 9 [42] was used for analyses.

### Permissions

No specific permissions were required for the work at the A.J. Powell Turf Research Center which is part of the Kentucky Agricultural Experiment Station's Spindletop Research Farm. Ryan Martin (Director of Horticulture, Gainesway Farm, Lexington, KY) granted access to the pesticide-free site for the bees to forage on the horse farm, and D. Davis (UK Facilities Management) granted access to the avoidance trial site. None of the field studies involved endangered or protected species.
